# Case Report: Petrous Apicitis and Otogenic Thrombosis of the Cavernous Sinus in a 10-Year-Old Boy

**DOI:** 10.3389/fsurg.2021.667817

**Published:** 2021-06-29

**Authors:** Patrick Bergsma, Seraina Kunz, Anna-Lena Kienle, Yves Brand

**Affiliations:** ^1^Department of Otolaryngology, Cantonal Hospital of Graubunden, Chur, Switzerland; ^2^Department Radiology, Cantonal Hospital of Graubunden, Chur, Switzerland; ^3^University Basel, Basel, Switzerland

**Keywords:** petrous apicitis, cavernous sinus thrombosis, otitis media, fusobacterium necrophorum, otomastoiditis

## Abstract

**Background:** Petrous apicitis and cavernous sinus thrombosis are exceedingly rare complications of acute otitis media with only few reported cases in the post-antibiotic era. Especially in children, the appropriate management is a subject of controversy.

**Case Presentation:** We report the case of a 10-year-old boy who presented to the emergency department with left-sided otalgia, otorrhea, and hearing loss, accompanied by somnolence and high spiking fevers. CT and MRI revealed partially obstructed mastoid air cells including a pneumatized petrous apex. Furthermore, thrombosis of the cavernous sinus and vasculitis of the internal carotid artery on the left side were present. The patient was treated with antibiotics for 6 weeks and anticoagulant therapy for 3 months. Follow-up carried out 3 months post-admission showed complete recanalization of the cavernous sinus on MRI and fatigue as the only remaining symptom.

**Conclusion:** A complicated otitis media with petrous apicitis and cavernous sinus thrombosis in a child can in some cases be managed without a surgical intervention.

## Introduction

Otitis media is the most common bacterial infection in children. Complications became rare in the era of antibiotics but can be life-threatening. They include acute mastoiditis, intracranial abscess, cranial nerve palsy, meningitis, petrous apicitis (PA) with or without osteomyelitis of the temporal bone, and otogenic cerebral venous sinus thrombosis (OCVST) (1, 2). Due to the anatomical proximity of the cerebral venous sinus system to the middle ear and mastoid air cells, infection, and inflammation can easily spread. A pneumatized petrous apex can lead to purulent PA and infections can reach the cavernous sinus. Reduced intravascular flow due to edema and a hypercoagulable state can lead to thrombosis (3). PA became an uncommon complication in the era of antibiotics and rarely affects children. It may present as Gradenigo Syndrome which describes the triad of otitis media, facial pain, and abducens palsy. Immediate administration of intravenous broad spectrum antibiotics is crucial. Further management of PA and OCVST regarding anticoagulation and surgical intervention remains controversial (1, 4, 5).

With our reported case we would like to present two rare complications of otitis media in the era of antibiotics and illustrate the radiological course under anticoagulant therapy.

Informed consent was obtained from the patient and his parents.

## Case Presentation

A 10-year-old otherwise healthy boy presented with left-sided otalgia and otorrhea since 2 days. Further he described hearing loss, somnolence and fever, peaking at 40°C.

Otoscopy revealed a perforated left-sided eardrum with purulent secretions. Retroauricular swelling and tenderness were present. Apart from marked somnolence, the neurological exam was normal with intact function of cranial nerves, including ocular motility.

### Diagnostic Assessment

**Figure 1:**
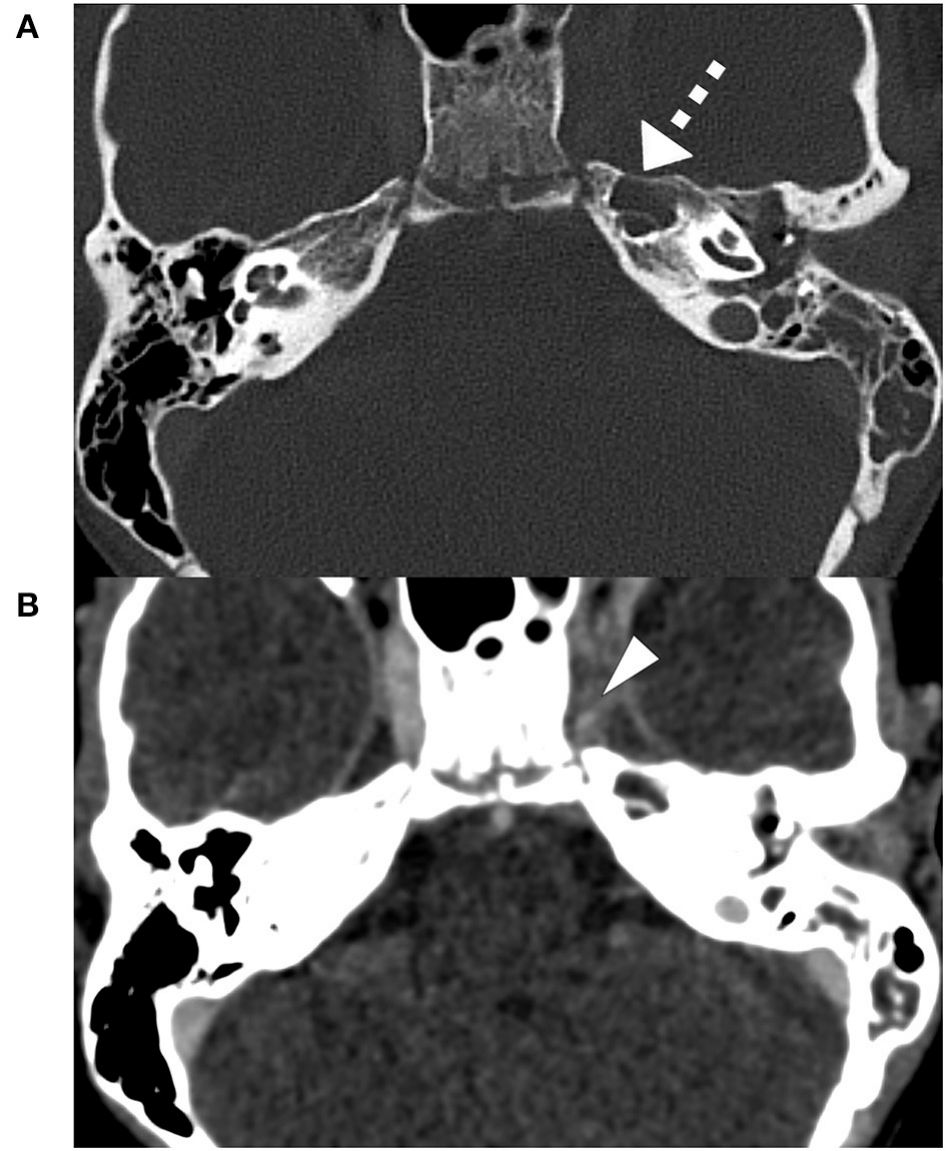
Initial contrast-enhanced CT-Scan bone window, slice thickness 0.75 mm: partially obstructed mastoid air cells with a fully opacified, pneumatized petrous apex (triangle) on the left side **(A)**. Initial contrast-enhanced CT-Scan soft tissue window, slice thickness 0.75 mm: Obstruction of the left cavernous sinus (dashed arrow), suspicious of cavernous sinus thrombosis **(B)**.

**Figure 2:**
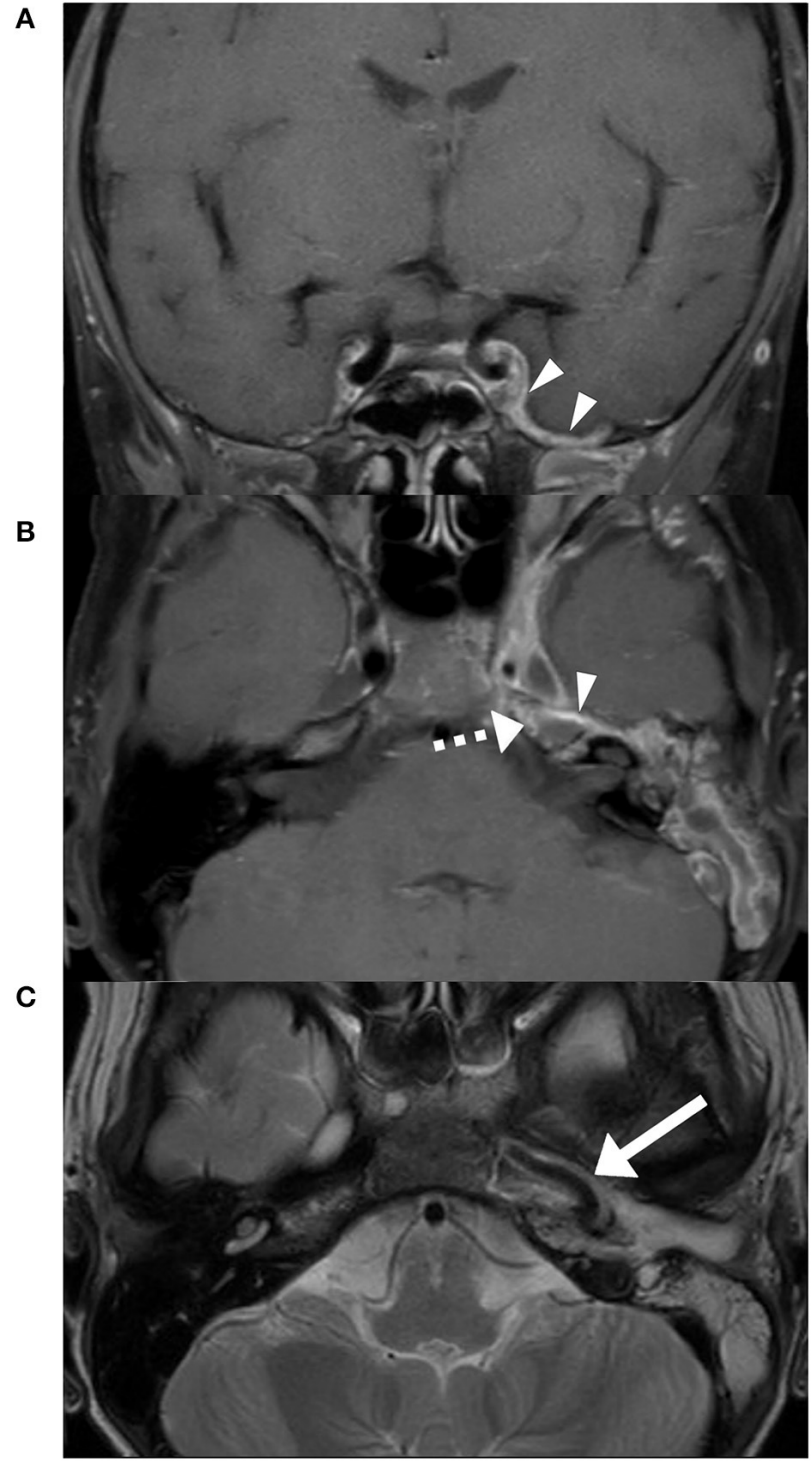
Initial MRI T1 DIXON FS post gadolinium. Dural enhancement (triangles) in the area of the left temporal lobe **(A)**. Anteromedial rim enhancement of the opacified petrous apex (dashed arrow) **(B)**. Thrombotic cavernous sinus and a marked vasculitis of the internal carotid artery (arrow) on the left side **(C)**.

**Figure 3:**
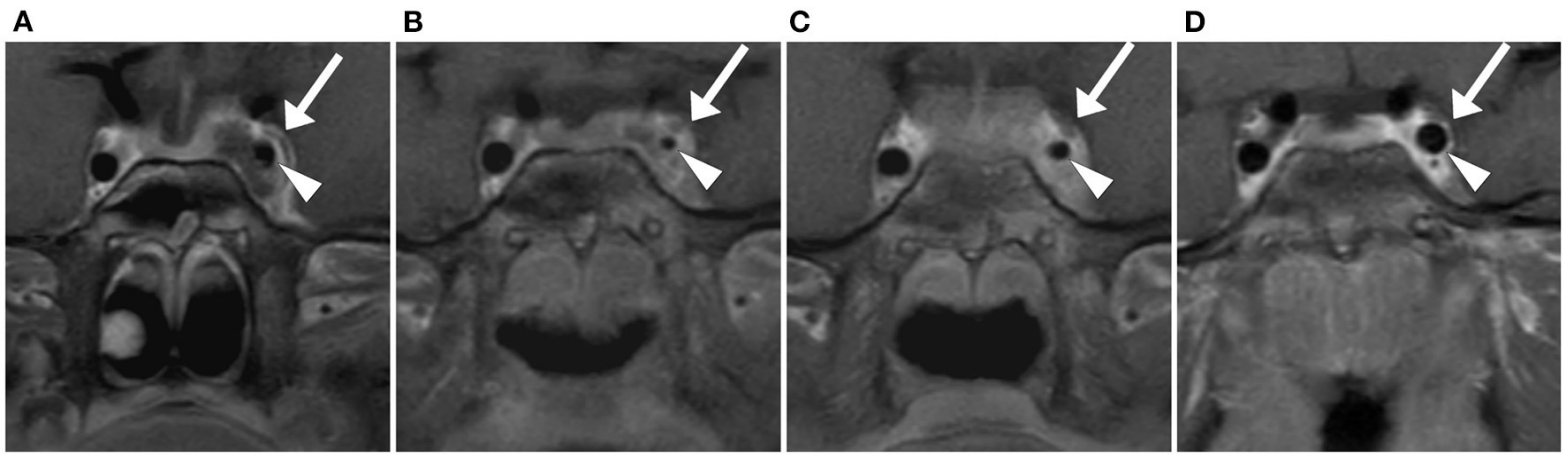
Coronal view T1 DIXON FS post gadolinium, slice thickness 3 mm. Arrow: Cavernous sinus, Triangle: Pars cavernosa of the internal carotid artery. Initial MRI: Thrombosis of the cavernous sinus and involvement of the internal carotid artery with hyperemia of the vasa vasorum **(A)**. MRI 7 days post-admission: Increasing inflammation of the internal carotid artery with further reduction in lumen size **(B)**. MRI 14 days post-admission: Improvement of internal carotid artery involvement with a partial recovery in size **(C)**. MRI 3 months post-admission: Complete recanalization of the left cavernous sinus and normal size of the left internal carotid artery **(D)**.

**Figure 4:**
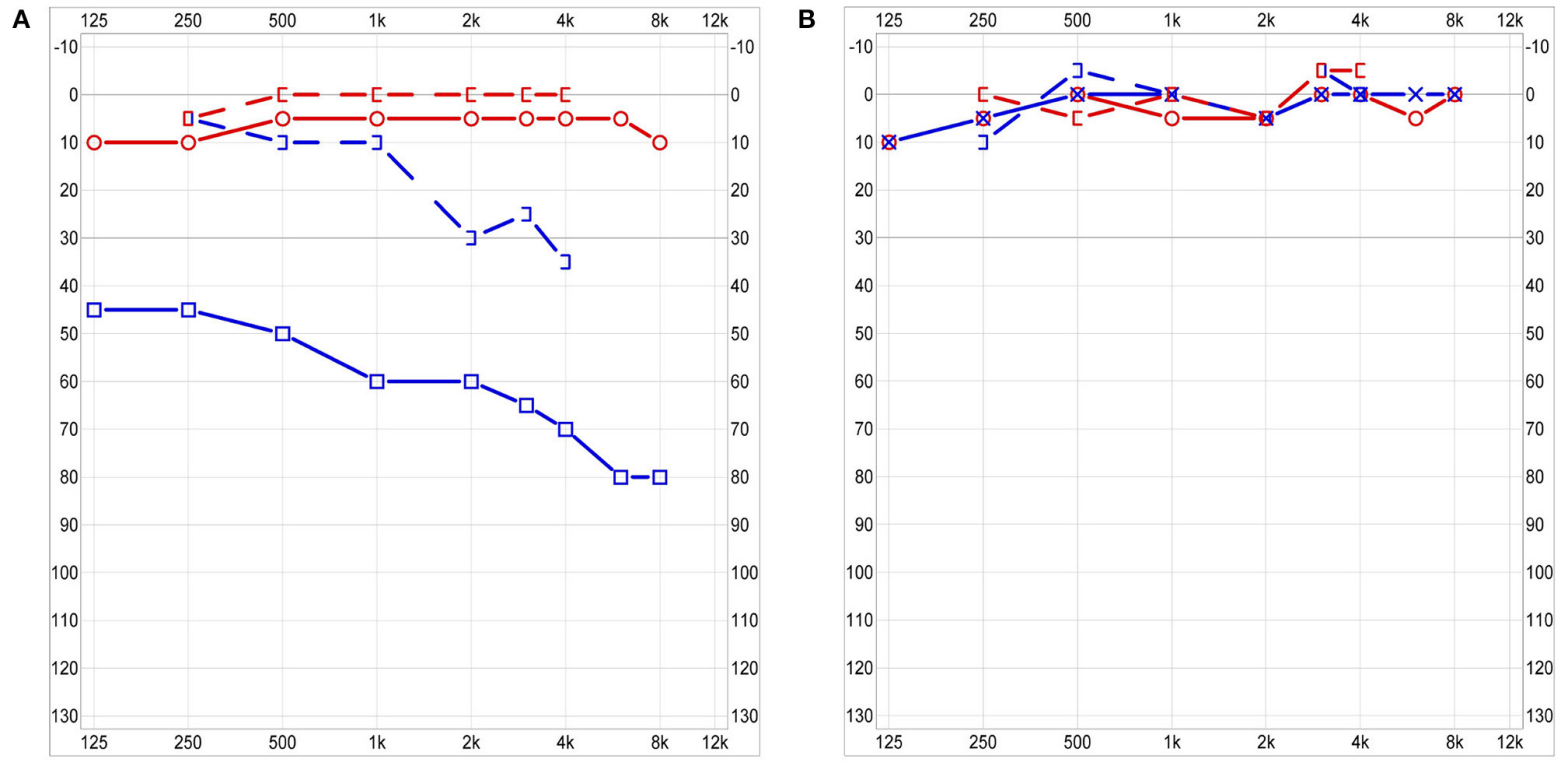
Audiogram 3 days post-admission: moderate to severe combined hearing loss on the left with normal hearing on the right side **(A)**. Audiogram at 3 months follow-up: Symmetrical normal hearing **(B)**.

A contrast-enhanced computed tomography (CT) revealed partially opacified mastoid air cells including a pneumatized petrosal apex on the left side and obstruction of the left cavernous sinus (Figure 1). Magnetic resonance imaging (MRI) showed rim enhancement in the opacified petrous apex and dural enhancement in the area of the left temporal lobe which suggested PA (Figures 2A,B). Furthermore, cavernous sinus thrombosis and vasculitis of the left-sided internal carotid artery were described (Figures 2C, 3A). A mixed, predominantly conductive hearing loss of 45–80 dB was present in pure tone audiogram on the left side in all tested frequencies. A small sensorineural aspect could be seen in the higher frequencies indicating a possible slight affection of the inner ear (Figure 4A). Lumbar puncture showed no signs of infection in the cerebrospinal fluid. *Fusobacterium necrophorum* was found in hemocultures.

### Treatment

Broad spectrum intravenous antibiotic therapy was administered immediately after presentation (single dose ceftriaxone, amoxicillin/clavulanic acid). After identifying *Fusobacterium necrophorum* in blood cultures, therapy was adjusted to metronidazole and ceftriaxone. Anticoagulant therapy with low weight molecular heparin, (enoxaparin sodium, 1 mg/kg subcutaneously every 12 h) was initiated immediately after radiological diagnosis. Since the perforated eardrum allowed for a constant clearance of middle ear purulence, no surgical intervention was performed.

In the first week of treatment, symptoms improved slowly. MRI was repeated 7 days post-admission and revealed a progressive thrombosis of the left cavernous sinus and vasculitis of the internal carotid artery (Figure 3B). Since symptoms improved and no new neurological findings occurred, no surgical intervention was considered. Fourteen days after hospitalization, a marked improvement of symptoms and reduced obstruction of cavernous sinus on MRI were present (Figure 3C). The patient could leave the hospital 27 days after hospitalization. Antibiotics were changed to clindamycin orally and continued until 6 weeks after initiation of treatment. Anticoagulant therapy was continued for 3 months. It was monitored by measuring coagulation factor Xa once a week.

### Follow-Up

Follow-up including MRI was carried out 3 months post-admission. Complete recanalization of the cavernous sinus and recovery of the internal carotid artery were shown and anticoagulant therapy could be stopped (Figure 3D). Apart from fatigue, patient described no remaining otological or neurological symptoms. A spontaneously healed, now intact eardrum with scarring was seen on otoscopy. Audiologic testing revealed normal symmetrical hearing with resolved conductive and sensorineural aspect of hearing loss (Figure 4B). A subcutaneous hematoma of about 8 cm in diameter in the area of injections of the anticoagulant drugs on the thigh was present. No further hemorrhagic complications were reported.

## Discussion

*Streptococcus pneumoniae* and *Haemophilus influenzae* are the predominant pathogens in acute otitis media amongst children and only a small percentage is caused by anaerobic bacteria. The cultured pathogen in the reported case was *Fusobacterium necrophorum*. The anaerobic gram-negative rod is traditionally known in the field of Otolaryngology as the most common pathogen in Lemierre‘s syndrome. An increased number of complicated otogenic infections caused by this bacteria have been reported in recent years. Recommended treatment consists of an antibiotic, targeting gram negative bacteria combined with a broad-spectrum beta-lactam antibiotic (6, 7).

Anticoagulation is widely used in context of cavernous sinus thrombosis in children. However, there is no significant evidence for positive effects on survival rate, cognitive outcome or recanalization rate (8). Anticoagulation therapy with low weight molecular heparin in OCVST seems relatively safe in children. In a systematic review by Wong et al., bleeding complications were reported in 7% of OCVST treated with anticoagulant drugs. They consisted of non-threatening hemorrhagic complications like epistaxis and postoperative hematoma. Only one out of 113 patients needed transfusion of red blood cells and fresh frozen plasma (4, 9). In our case we observed a hematoma in the area of anticoagulant injections, which can be considered a mild hemorrhagic complication. There is no general recommendation on duration of anticoagulant therapy. It varies from 3 weeks to 12 months (9, 10).

The necessity of surgery in OCVST is a subject of controversy. Described surgical procedures vary widely in their extent. They include more conservative approaches like myringotomy with tube insertion and mastoidectomy. Exploration of the sigmoid sinus, thrombectomy, resection of the affected sinus, and ligation of the internal jugular vein represent more extensive procedures. A trend toward more conservative techniques has been observed in recent years (11–13). About 10% of cases are managed without a surgical intervention at all (4). In our review of the literature we only found two cases of OCVST affecting the cavernous sinus in children. One case is described in a case series by Ulanovski et al. where the management of this specific case is not further explained. A second case we found in a case series by Neilan et al., where the cavernous sinus thrombosis was accompanied by thrombosis of the internal jugular vein and sigmoid sinus. Mastoidectomy, tympanostomy with grommet insertion and decompression of the sigmoid sinus with a needle was performed (11, 12). In our case, the infection spread to the cavernous sinus through an opacified pneumatized petrous apex which is one of the most challenging areas to access in skull base surgery. Possible interventions are extensive, technically difficult, and accompanied by a considerable risk of complications. They include a transmastoid approach following fistulous tracts around vital structures, the translabyrinthine approach, transsphenoid approaches, and the middle cranial fossa approach. Gadre et al. suggested immediate initiation of empiric intravenous antibiotic treatment and reconsideration of operative treatment if there is no improvement after 24–48 h (1).

With radiologically only partially obstructed mastoid air cells and spontaneous drainage of middle ear purulence, broad-spectrum antibiotics, anticoagulation, and no surgical intervention led in our reported case to a very positive outcome. Complete recanalization of the affected cerebral sinus on MRI (Figure 3D) and apart from fatigue, no symptoms were present 3 months post-admission.

## Conclusion

With our presented case, we hope to highlight that even with rare and threatening complications of otitis media such as PA and cavernous sinus thrombosis, surgical intervention is not always a necessity. Since these complications are exceedingly rare, there is no consent on its management in children. Prospectively collected data would be needed to define evidence-based guidelines on surgical and anticoagulant treatment. Since feasibility of high level evidence studies is questionable, reports of single cases remain important in the discussion of best possible care.

### Perspective of the Patient's Mother

“We were shocked when our son had to be hospitalized. We've never seen him in a state like that. Even though we're very happy he's doing much better again, we're worried something similar could happen.”

## Data Availability Statement

The raw data supporting the conclusions of this article will be made available by the authors, without undue reservation.

## Ethics Statement

Written informed consent was obtained from the minor(s)' legal guardian/next of kin for the publication of any potentially identifiable images or data included in this article.

## Author Contributions

YB was the main treating physician, initiated the study, supervised the work, and revised the manuscript. PB and SK drafted the manuscript. A-LK described the radiological findings. All authors contributed to the article and approved the submitted version.

## Conflict of Interest

The authors declare that the research was conducted in the absence of any commercial or financial relationships that could be construed as a potential conflict of interest.
