# Supplementary material for: Case Report: Petrous Apicitis and Otogenic Thrombosis of the Cavernous Sinus in a 10-Year-Old Boy
Source: Front Surg. 2021 Jun 29;8:667817. doi: 10.3389/fsurg.2021.667817 (PMC8275649; doi:10.3389/fsurg.2021.667817)
Supplement: Supplementary file 1 [file Data_Sheet_1.PDF]

CARE Checklist of information to include when writing a case report

CARE case report guidelines

(cc) BY-NC-ND

Topic	Item	Checklist item description	Reported on Line
Title	1	The diagnosis or intervention of primary focus followed by the words "case report"	1-2
Key Words	2	2 to 5 key words that identify diagnoses or interventions in this case report, including "case report"	9-10
Abstract (no references)	3a	Introduction: What is unique about this case and what does it add to the scientific literature?	12-14
	3b	Main symptoms and/or important clinical findings	15-17
	3c	The main diagnoses, therapeutic interventions, and outcomes	17-21
	3d	Conclusion—What is the main "take-away" lesson(s) from this case?	22-23
Introduction	4	One or two paragraphs summarizing why this case is unique (**may include references**)	25-36
Patient Information	5a	De-identified patient specific information.	41
	5b	Primary concerns and symptoms of the patient.	41-42
	5c	Medical, family, and psycho-social history including relevant genetic information	41
	5d	Relevant past interventions with outcomes	-
Clinical Findings	6	Describe significant physical examination (PE) and important clinical findings.	43-45
Timeline	7	Historical and current information from this episode of care organized as a timeline	-
Diagnostic Assessment	8a	Diagnostic testing (such as PE, laboratory testing, imaging, surveys).	47-54
	8b	Diagnostic challenges (such as access to testing, financial, or cultural)	-
	8c	Diagnosis (including other diagnoses considered)	47-52
	8d	Prognosis (such as staging in oncology) where applicable	-
Therapeutic Intervention	9a	Types of therapeutic intervention (such as pharmacologic, surgical, preventive, self-care)	56-59
	9b	Administration of therapeutic intervention (such as dosage, strength, duration)	60,66
	9c	Changes in therapeutic intervention (with rationale)	58, 66-67
Follow-up and Outcomes	10a	Clinician and patient-assessed outcomes (if available)	64-65, 69-75
	10b	Important follow-up diagnostic and other test results	69-75
	10c	Intervention adherence and tolerability (How was this assessed?)	-
	10d	Adverse and unanticipated events	73-74
Discussion	11a	A scientific discussion of the strengths AND limitations associated with this case report	78-81, 90-91, 112-115
	11b	Discussion of the relevant medical literature **with references**.	84-90,97-111
	11c	The scientific rationale for any conclusions (including assessment of possible causes)	112-114
	11d	The primary "take-away" lessons of this case report (without references) in a one paragraph conclusion	120-125
Patient Perspective	12	The patient should share their perspective in one to two paragraphs on the treatment(s) they received	-
Informed Consent	13	Did the patient give informed consent? Please provide if requested	Yes ☑ No ☐
